# Correction: Resource plasticity-driven carbon-nitrogen budgeting enables specialization and division of labor in a clonal community

**DOI:** 10.7554/eLife.74046

**Published:** 2021-09-23

**Authors:** Sriram Varahan, Vaibhhav Sinha, Adhish Walvekar, Sandeep Krishna, Sunil Laxman

Varahan S, Sinha V, Walvekar A, Krishna S, Laxman S. 2020. Resource plasticity- driven carbon-nitrogen budgeting enables specialization and division of labor in a clonal community. *eLife* **9**:e57609. doi: 10.7554/eLife.57609.

Published 2, September 2020

In the published article, the colony images shown in Figure 1 are intentionally reused in Figure 1-figure supplement 1, Figure 2, Figure 4 and Figure 5, in order to provide a consistent representation of the data for the readers (without having to refer back to Figure 1). These are representative images from experiments that were done in triplicate (unless indicated otherwise). Additionally, in the schematic experimental flow diagram in Figure 2C, the two colony images (from Figure 1) are reused solely for illustrative purposes.

We apologize that the intentional reuse of images in multiple panels for illustration purposes was not made clear in the figure legends.

